# Whole-Plant Dynamic System of Nitrogen Use for Vegetative Growth and Grain Filling in Rice Plants (*Oryza sativa* L.) as Revealed through the Production of 350 Grains from a Germinated Seed Over 150 Days: A Review and Synthesis

**DOI:** 10.3389/fpls.2016.01151

**Published:** 2016-08-03

**Authors:** Tadakatsu Yoneyama, Fumio Tanno, Jiro Tatsumi, Tadahiko Mae

**Affiliations:** ^1^Department of Applied Biological Chemistry, The University of TokyoTokyo, Japan; ^2^Fukushima Prefecture Kennan Agricultural and Forestry OfficeFukushima, Japan; ^3^Kyoto Laboratory of Eco-PlantsKyoto, Japan; ^4^Graduate School of Agricultural Science, Tohoku UniversitySendai, Japan

**Keywords:** amino acids, grain-filling, nitrogen assimilation, phloem transport, protein synthesis and degradation, remobilization, rice (*Oryza sativa* L.), vegetative growth

## Abstract

A single germinated rice (*Oryza sativa* L) seed can produce 350 grains with the sequential development of 15 leaves on the main stem and 7–10 leaves on four productive tillers (forming five panicles in total), using nitrogen (N) taken up from the environment over a 150-day growing season. Nitrogen travels from uptake sites to the grain through growing organ-directed cycling among sequentially developed organs. Over the past 40 years, the dynamic system for N allocation during vegetative growth and grain filling has been elucidated through studies on N and ^15^N transport as well as enzymes and transporters involved. In this review, we synthesize the information obtained in these studies along the following main points: (1) During vegetative growth before grain-filling, about half of the total N in the growing organs, including young leaves, tillers, root tips and differentiating panicles is supplied via phloem from mature source organs such as leaves and roots, after turnover and remobilization of proteins, whereas the other half is newly taken up and supplied via xylem, with an efficient xylem-to-phloem transfer at stem nodes. Thus, the growth of new organs depends equally on both N sources. (2) A large fraction (as much as 80%) of the grain N is derived largely from mature organs such as leaves and stems by degradation, including the autophagy pathway of chloroplast proteins (e.g., Rubisco). (3) Mobilized proteinogenic amino acids (AA), including arginine, lysine, proline and valine, are derived mainly from protein degradation, with AA transporters playing a role in transferring these AAs across cell membranes of source and sink organs, and enabling their efficient reutilization in the latter. On the other hand, AAs such as glutamine, glutamic acid, γ-amino butyric acid, aspartic acid, and alanine are produced by assimilation of newly taken up N by roots and and transported via xylem and phloem. The formation of 350 filled grains over 50 days during the reproductive stage is ascribed mainly to degradation and remobilization of the reserves, previously accumulated over 100 days in the sequentially developed vegetative organs.

## Introduction

**FIGURE 1:**
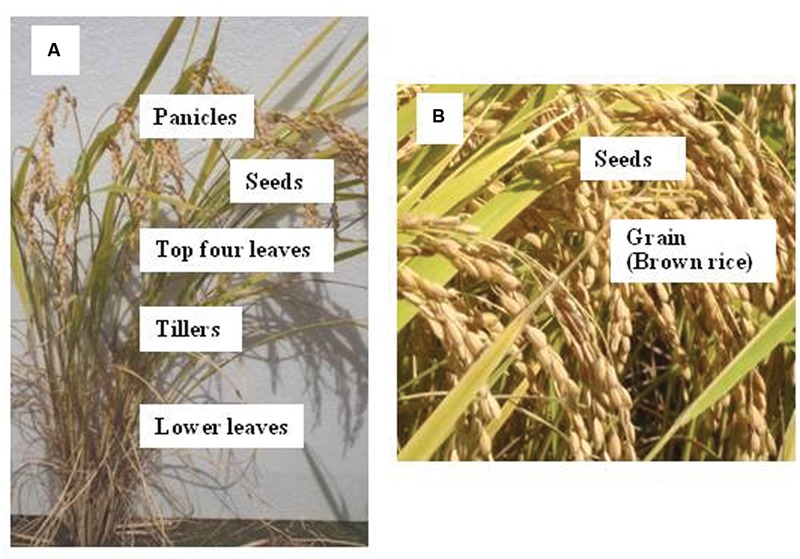
**Pictures showing a hill **(A)** and canopy **(B)** of Koshihikari, a high eating-quality rice, at their harvest (5,400 kg ha^-1^ of brown rice) in the Fukushima district, Japan.** Pictures taken on September 29, 2015.

Rice (*Oryza sativa* L.) is a member of the Graminaceae, which also include wheat (*Triticum aestivum* L.) and corn (*Zea mays* L.). These plant species develop a main stem with tillers and finally panicles (called kernels in corn) containing grains. In a paddy field of Fukushima prefecture, Japan, a single hill cultivated with four rice seedlings (cv. Koshihikari, high eating-quality rice) was reported to produce 1,400 grains (**Figure 1**). During a 70-day vegetative stage, a single seedling develops a main stem with 11 leaves and approximately 4 productive tillers with 4–6 leaves each. Then, during the 32 days of stem-internode elongation before heading, the main stem and each tiller develop four additional leaves including the flag leaf at the top, and five panicles. Finally, over a 50-day period following elongation of the leaves, 350 (70 × 5; both in the main stem and four tillers) grains per plant are filled in the panicles. In some of the high-yielding rice cultivars, the number of grains per plant could be up to twofold higher (Mae, 2011).

Koshihikari rice plants take up nitrogen (N) from the environment (paddy-soil fields) during the whole 150-day growth period to develop shoots, roots, and tillers, and specific partitioning of N between plant parts occurs at any growth stage. To elucidate N dynamics in rice, intensive analyses of N accumulation among rice organs have been conducted using ^15^N as a tracer, but the resulting information is still fragmentary due to the lack of theoretical assessments for inter-organ N partitioning. In this review, we explore the mechanisms by which N contribute to vegetative growth and grain filling, by identifying the source and sink relationships for N and describe the dynamic state of the N reserves and N uptake in rice. We describe for the first time the whole features of the N dynamics at the whole-plant level that allow for the production of 350 rice seeds from a single germinated one.

In the first part of this review we describe the mechanisms of N uptake, assimilation and metabolism in rice, with special consideration to N partitioning and the movement of individual amino acids (AA) facilitated by transporters through the vascular systems during the development of different organs.

## Current Knowledge on the Uptake, Assimilation, and Transport of Nitrogen in Rice Plants

### Nitrogen Uptake and Assimilation in the Roots

Rice plants grown in soils take up mostly ammonium under flooded conditions and nitrate under drained conditions via their respective transporters. Then, N is assimilated into AAs by using a series of enzymes, including nitrate and nitrite reductases, glutamine synthetase, glutamate synthase, etc., to build up proteins, nucleic acids and other N-containing constituents (Yoneyama et al., 2003).

**FIGURE 2:**
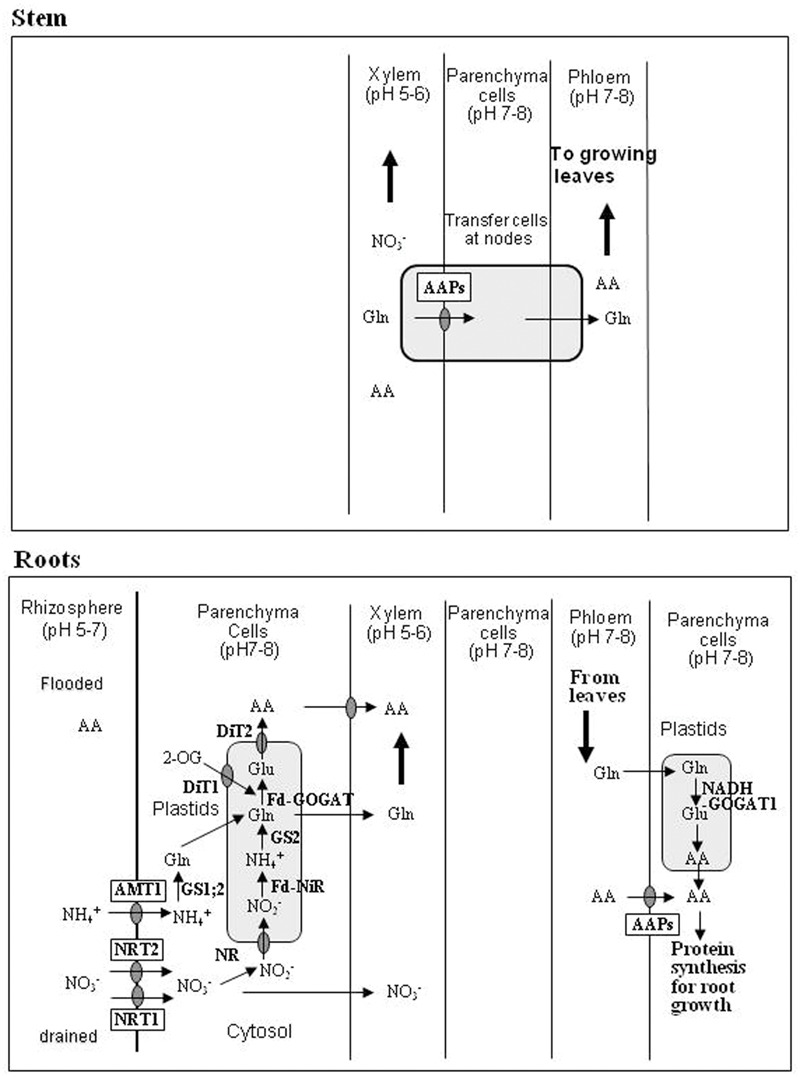
**Models for uptake, assimilation, and transport of N in stem and roots of rice.** Abbreviations: AA, amino acids; AAPs, amino acid permeases; DiT1, 2-oxoglutarate translocator; DiT2, glutamate translocator; NRT1, low-affinity nitrate transporter; NRT2, high-affinity nitrate transporter.

In flooded soils, ammonium is released from soil and plant residue organic N and supplemented in the form of chemical fertilizers (in Japan, this is done mostly with ammonium sulfate and urea). In rice roots, ammonium is taken up by the ammonium transporters (OsAMT1s) expressed in the root cell plasma membranes (Sonoda et al., 2003) (**Figure 2**). ^15^N-labeled ammonium added to the root bathing culture solution is fully assimilated first into glutamine and then into glutamate in the roots (Yoneyama and Kumazawa, 1974). The cytosol ammonia is assimilated to the amide of glutamine by cytosolic glutamine synthetase 1 (GS1, EC 6.3.1.2) in rice roots (Hirel and Gadal, 1980; Funayama et al., 2013) and then, glutamine is transformed through the GS-GOGAT pathway to glutamic acid either by the ferredoxin-dependent glutamate synthase (Fd-GOGAT, EC 1.4.7.1; Suzuki et al., 1982) or by the NADH-dependent glutamate synthase (NADH-GOGAT1, EC 1.4.1.14; Yamaya et al., 1995). Both enzymes are located in plastids in root cells. ^15^N-labeling of asparagine from ^15^N-ammonium occurs steadily in the rice roots (Yoneyama and Kumazawa, 1974), and this is probably catalyzed by the glutamine-dependent asparagine synthetase 1 (OsAS1, EC 6.3.5.4; Kawachi et al., 2002; Ohashi et al., 2015). When rice seedlings were treated with ^15^N-labeled ammonium sulfate, a rapid labeling of glutamine and a steady labeling of asparagine were detected in the xylem exudates (Yoneyama, 1986).

Nitrate in the soil solution is taken up into root cells by the high-affinity nitrate transporter (NRT2) in low-nitrate medium and the low-affinity nitrate transporter (NRT1) in high-nitrate medium (Hasegawa, 1996; Lin et al., 2000) (**Figure 2**). ^15^N-labeled nitrate taken up by rice seedling roots is transformed to ammonium, glutamine, glutamate and other AAs (Yoneyama and Kumazawa, 1975), probably first by nitrate reduction to ammonia and subsequently by the GS-GOGAT pathway. Part of the nitrate in the cytosol of root cells is reduced to nitrite by the NADH-dependent nitrate reductase (NADH-NR; EC 1.6.6.2), and the nitrite formed is imported into plastids to be reduced to ammonia by the Fd-dependent nitrite reductase (Fd-NiR; EC 1.6.6.4). Ammonia in the plastids can be assimilated by glutamine synthetase 2 (GS2; EC 6.3.1.2), with the formed glutamine being further transformed to two glutamates by the Fd-GOGAT (EC 1.4.7.1; Suzuki et al., 1982). Unreduced nitrate is partly transported to the shoots via xylem.

Here, it is worth noting the recent finding that the phytohormone cytokinin effectively functions under nitrate and ammonium nutrition in rice plants. Cytokinin accumulated in the xylem sap following nitrate or ammonium treatment on rice roots, and the synthesis of cytokinins occurred in parallel with enhanced gene expression of adenosine phosphate-isopentenyltransferase 4 (*IPT4*) by glutamine or a related metabolite, produced during nitrate or ammonium assimilation (Kamada-Nobusada et al., 2013).

Roots (particularly the growing root tips) also receive AAs and amides from mature shoot organs via phloem to synthesize proteins for root growth (Tatsumi and Kono, 1980a). During such processes, glutamine unloaded from the phloem may be metabolized by NADH-GOGAT leading to the synthesis of glutamate (Hayakawa et al., 1999), which is utilized to initiate the synthesis of various AAs.

Rice roots export the assimilated AAs and unreduced nitrate to the aerial parts via the xylem in the stem (Yoneyama, 1986) (**Figure 2**). Here, N chemical form-dependent differences in partitioning are noteworthy: ^15^N fed to the roots as ammonium is actively transported to the growing leaves, whereas ^15^N fed to the roots as nitrate is transported mainly to the mature leaves apparently following the xylem transpiration stream (Yoneyama and Kumazawa, 1972). Such differential transport may be ascribed to the N chemical forms ascending in the xylem. The ammonium fed to rice roots is almost totally assimilated into glutamine with a smaller amount being further converted to asparagine (Yoneyama, 1986), and such AAs are preferentially transferred from xylem to phloem at the stem nodes, as revealed by a positron emitting tracer imaging system (PETIS, Kiyomia et al., 2001). The stem nodes of rice plants contain xylem transfer cells (Zee, 1972; Kawahara et al., 1974), which are involved in the xylem-to-phloem transport of AAs mediated by amino acid permeases (AAPs) (Tegeder and Ward, 2012; Taylor et al., 2015). However, this xylem-to-phloem transfer does not occur for anionic nitrate, which is only transported via xylem.

A preferential way of transport of the ^15^N derived from ^15^N-labeled dinitrogen or ammonium in soybean (*Glycine max*) plants consists in assimilation into ureides and AAs in the underground part of the plant and the subsequent transport to the developing organs, young leaves and developing pods, as compared to the transpiration-dependent transport of nitrate-^15^N (Yoneyama and Ishizuka, 1982). Also, feeding of ^14^C-labeled AAs into the transpiration stream through the cut stem bases of young tomato plants resulted in preferential accumulation to younger leaves: ^14^C-labeled AAs were transported by xylem-to-phloem transfer, whereas inulin [^14^C]carboxylic acid, which was used as a reference for xylem transport, was distributed to mature leaves depending on the transpiration rates of the leaves (van Bel, 1984).

### Nitrogen Metabolism in the Shoots

**FIGURE 3:**
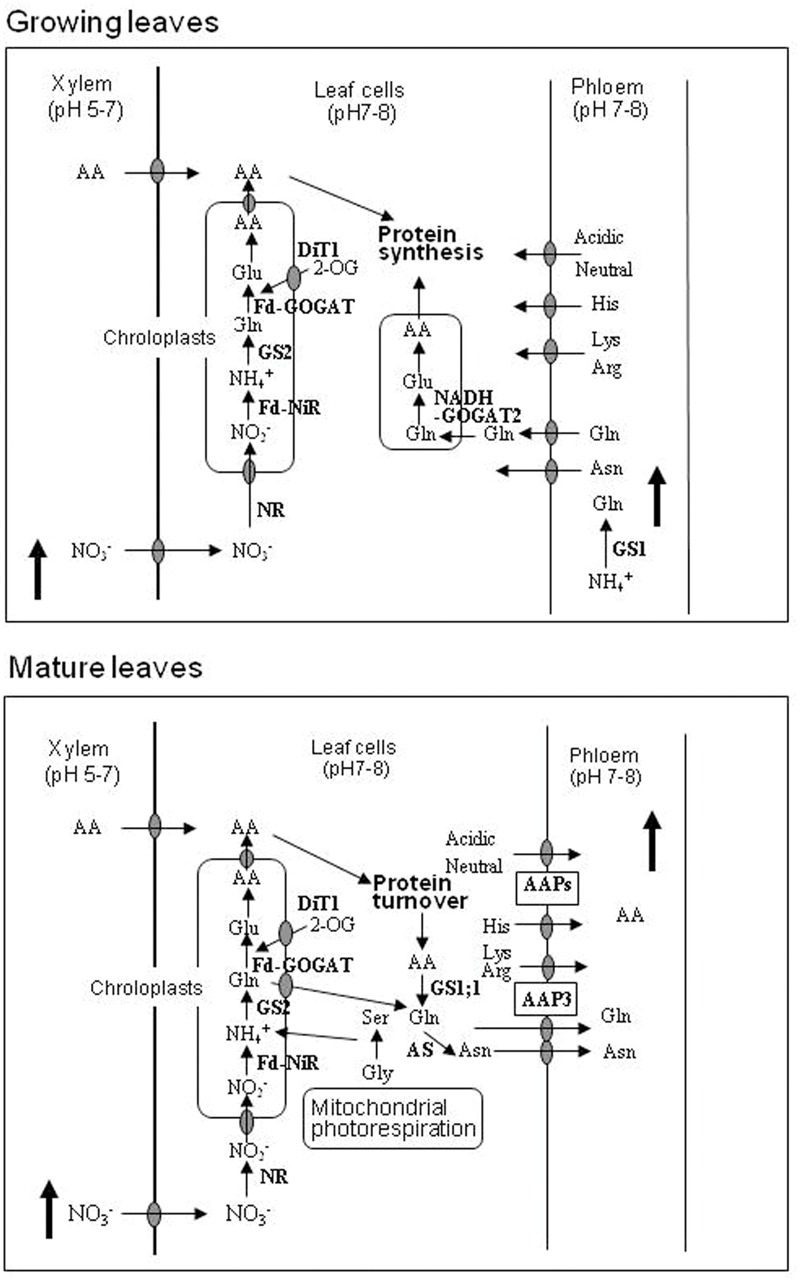
**Models for assimilation, metabolism, and transport of N in growing and mature leaves in rice**.

Leaves can reduce nitrate to ammonium and assimilate it to glutamine and glutamate under light and dark conditions, with light accelerating nitrite reduction and glutamate formation due to an efficient supply of electrons from Photosystem I (**Figure 3**). Plants develop and synthesize membrane structures and proteins (transporters and enzymes) to drive transport of inorganic nutrients and metabolites targeting at the meristems to produce new organs and tissues. AA transporters have been shown to participate in the loading of AAs to the phloem (Tegeder and Rentsch, 2010; Tegeder and Ward, 2012).

The analysis of AA fluxes in mature leaves of rice seedlings indicates that the export of AAs occurs via phloem after protein turnover (Yoneyama and Takeba, 1984). The export of re-mobilized N terminates at the growing leaves (Yoneyama and Sano, 1978). The AAs exported in the phloem (Fukumorita and Chino, 1982) include: (i) actively metabolized AAs and amides, such as glutamine, glutamic acid, asparagine, aspartic acid, and alanine, which are derived from inorganic N assimilation and also produced after active interconversion in the source organs, and (ii) more importantly, coordinately regulated AAs such as lysine, arginine, histidine, valine, tyrosine, phenylalanine, and leucine (Noctor et al., 2002), which are synthesized in the chloroplast under the tight control of feedback-regulation (Pratelli and Pilot, 2014) and are also supplied after protein degradation.

The phloem saps from the flag-leaf sheath and uppermost internode in rice plants contained glutamine as much as 18 and 42% of the total AAs, respectively (Hayashi and Chino, 1990). This increase of glutamine percentages may be associated with the GS1 activity detected in the rice phloem sap, which contains not only GS protein but also the substrates (${\mathrm{NH}}_{4}^{+}$, ATP, and glutamate) (Tanaka et al., 2009). Cytosolic glutamine synthetase (GS1, Kamachi et al., 1992) in the companion cells near the phloem sieve cells in the leaf midribs was demonstrated to be important to sustain glutamine concentrations in the phloem sap. On the other hand, in sink organs such as unexpanded non-green leaves (Yamaya et al., 1992) and young spikelets (Hayakawa et al., 1993), there is a high activity of NADH-GOGAT1, allowing for the synthesis of glutamate from glutamine imported via phloem from the reserves (**Figure 3**).

The nitrogenous compounds in the phloem sap can act as indicators of the plant N status and constitute a signal to control root N uptake using a biochemical feedback system (Britto and Kronzucker, 2004). The phloem transport of N compounds must be fully controlled to sustain the new growth of the organs and to efficiently respond to N demands by balancing internal remobilization and exogenous root uptake: the demand of the sink organs must be adequately sensed, and certain long-distance signals must work in this sensing-signaling system. Nitrate was detected in the rice phloem sap at a concentration of 1.9 mM when the nutrient solution contained 0.35 mM nitrate (Hayashi and Chino, 1985), and an interesting function of the vein phloem-localized nitrate transporters (NRT1s) in the redistribution of xylem-borne nitrate was found in *Arabidopsis* (Hsu and Tsay, 2013). However, the sensing and signaling system permitting the optimization plant growth is still largely unknown. Considerable amounts of lysine and threonine have been detected in the phloem sap (Fukumorita and Chino, 1982): such phloem-delivered AAs may suppress the new synthesis of these AAs in sink organs via a feed-back mechanism, likely leading to their incorporation into new proteins in the sink organs without further transformation.

Amino acids transported upward in the stem are finally accumulated in the panicles, after unloading via membrane-localized AA transporters (Tegeder and Ward, 2012), in order to produce seeds for the next generation. In the seeds, a large fraction (about 50%) of the imported AAs is used to synthesize storage proteins (glutelin, prolamin) that accumulate in protein bodies (Shewry and Halford, 2002).

## Organ Growth is Sustained By Both the Amino Acids Re-Mobilized From Endogenous Nitrogen Reserves in Mature Organs and those Synthesized From Exogenous Nitrogen Sources

### Importance of Reserve N for Growth of Shoot Organs

**FIGURE 4:**
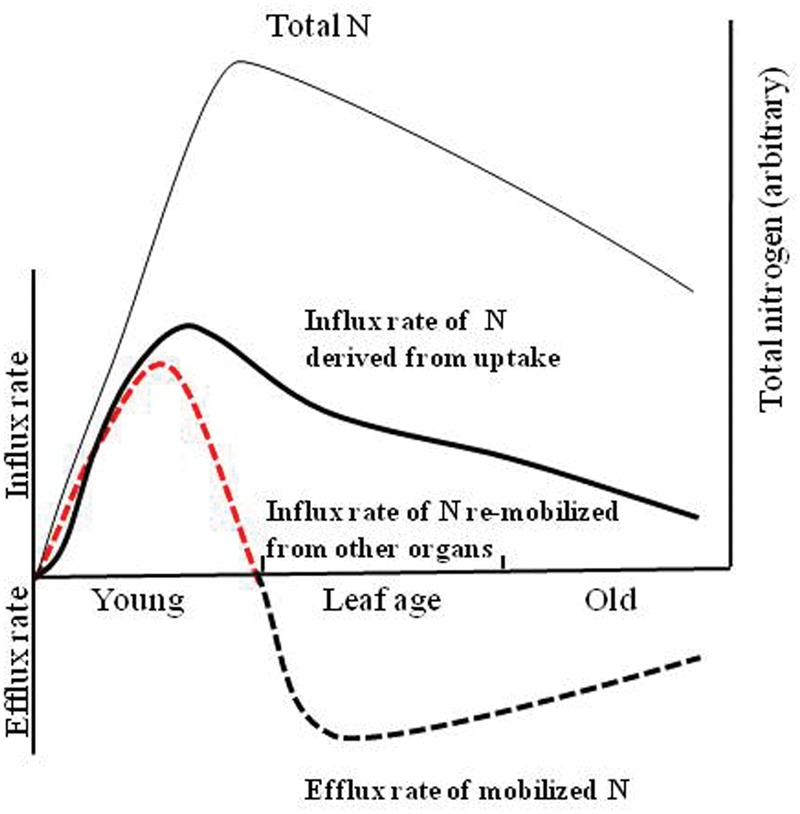
**Modeling of the influx rates of N re-mobilized from other organs and N derived from uptake and eﬄux rate of mobilized N with reference to the total N content of a leaf.** Adapted from Yoneyama and Sano (1978).

In rice seedlings, ^15^N partitioned to mature leaves was shown to re-translocate to still growing ones, including the leaf which started to grow 1 week after the ^15^N feeding event (Yoneyama, 1977). Yoneyama and Sano (1978) first examined N accumulation in the growing leaves of rice seedlings at the 5th leaf growth stage by quantifying N newly taken up using a ^15^N tracer under the assumption that the remaining N (non-labeled) would come from internal reserve sources. Based on these analyses, the authors proposed a model for N: half of the total N required for the growth of new organs was re-translocated by remobilization of the reserves, with the remaining half being supplied by uptake of exogenous N (**Figure 4**). At the 90% maximum N-accumulation, endogenous influx from re-mobilized N became smaller and the own leaf-N was apparently the N source for export to the other growing organs. In the mature leaf, the import of the newly absorbed N and export of re-mobilized N make balance. The import of the newly taken up N decreases with age, if additional N fertilization is not applied, and the decrease of the N content of mature leaves proceeds in parallel. At leaf senescence, the relative eﬄux rate becomes larger when compared to the influx rate. As stated above, some of the xylem AAs may be efficiently transferred to phloem by the xylem-to-phloem system at node-located transfer cells; therefore, a major route for N influx for the growth of new organ is via the phloem. A quantitative study using a ^15^N-tracing technique indicated that the N in the emerging 12th-leaf blades came nearly 50% (63 and 45%) by remobilization from older parts (including mature leaves, stem, and roots) with the rest coming from newly taken up ^15^N-labeled ammonium (Mae and Ohira, 1981).

The contributions of newly absorbed ammonium and internally re-mobilized N as sources for individual AAs was unveiled by separately labeling with ^15^N at different times the currently taken up N and the re-mobilized N (**Table 1**, Yoneyama, 1978a). Glutamic acid, glutamine, γ-amino butyric acid, aspartic acid, and alanine were those with the higher percentages of the newly taken up N. Regarding re-mobilized N, arginine, proline, lysine, and asparagine in free AAs and arginine, lysine, proline, and valine in the bound AAs had the high percentages of N derived from remobilization in the source organs. Similar differential contributions of seed-derived N and newly taken up N were found in individual AAs in young leaves and roots of rice, corn and soybean: phenylalanine, tyrosine, proline, lysine and valine were relatively more dependent on the N seed reserve whereas glutamine, asparagine, γ-amino butyric acid, glutamic acid and alanine were more dependent on current uptake (Yoneyama, 1978b). When ^14^C-labeled serine, lysine, and leucine were applied to a fully expanded source leaf of oat (*Avena sativa* L.), lysine and leucine were transported to the immature sink leaf via phloem without any metabolic change, whereas serine was extensively metabolized in the source leaf (Peterson et al., 1977). A differential contribution of the early taken up and currently re-mobilized N and the newly taken up N was observed in AAs of grain proteins in nitrate-grown wheat: lysine, valine, proline, leucine + isoleucine, and arginine + histidine were derived from the former N source and glutamic acid, aspartic acid, and alanine from the latter (Yoneyama, 1983). When ^14^C-labeled lysine was injected to the uppermost internode of ripening wheat, 46–49% of it was found as lysine in the ripened grains, suggesting that the lysine preformed elsewhere in the plant was transported and incorporated into the grain protein (Lawrence and Grant, 1964). It is worth noting that spectrum of AAs re-mobilized from the reserve organs and re-utilized in the growing organs were apparently in accordance with that of AAs, which were reportedly to be highly coordinated in the leaves (Noctor et al., 2002) and that these AAs are mostly essential AAs for humans and therefore important for the grain AA composition.

**Table 1:** Percentages of N in free and bound amino acids (AA) in youngest 6^th^ leaf either derived from the ammonium taken up for 24 h at the 4^th^ leaf growth at day 22 (Re-mobilized N) or from ammonium taken up for 10 h just before harvest (Currently taken up N] at day 31.

Amino acid	Currently taken up N		Re-mobilized N	
	Free AA	Bound AA	Free AA	Bound AA
Glutamic acid	30.2	4.58	1.22	0.86
Glutamine	29.2		1.46	
γ-Amino butyric acid	29.8		1.48	
Aspartic acid	27.6	3.70	1.32	0.94
Alanine	25.8	3.32	1.34	0.84
Valine	21.4	1.68	1.00	1.38
Tyrosine	14.4	1.50	1.44	1.14
Asparagine	12.5		1.62	
Serine	12.3	ND	1.30	ND
Glycine	12.1	ND	1.32	ND
Phenylalanine	10.8	1.78	1.28	1.16
Proline	10.5	1.58	1.66	1.30
Arginine	10.3	2.26	2.00	1.94
Lysine	7.6	1.88	1.50	1.70
Leucine + Isoleucine	6.9	2.06	1.34	1.20

*N.D., not determined separately. Data from Yoneyama (1978a).*

The importance of the stem N content for the development of tiller primordia and the increase in tiller numbers was demonstrated by Tanaka and Garcia (1965). When the roots of primary tillers were fed with ^15^N-labeled ${\mathrm{NH}}_{4}^{+}$, ^15^N was mainly transported to the growing secondary tillers produced from the primary ones, whereas a smaller ^15^N transfer to the main stem and primary tillers was found (Mimoto et al., 1990). Thus, both the N stored in the stems and leaves and the newly taken up N may be effective for tiller development.

### Contribution of Reserve N to the Growth of Roots

Direct foliar application of ^15^N-labeled urea solution for 16 h per day over 7 days separately to two different mature leaves in the main stem, either the mature 8th leaf blade or the old 5th leaf blade, was investigated in terms of the ^15^N distribution in the whole plant (Tatsumi and Kono, 1981). The ^15^N from both leaves was actively partitioned to the growing 9^th^ and even more actively to the 10th leaves, as well as to the growing tillers, whose development occurred in the 6th and 7th nodes, respectively. The contribution of ^15^N to the growing roots occurred at the same rates with and without supply of ammonium to the solution bathing the roots. The proportions of N translocated from the other plant parts into the growing leaves and roots were estimated to be 46–52% and about 59%, respectively.

When the 12th-leaf-age rice plants (with tillers removed) were fed with ^15^${\mathrm{NH}}_{4}^{+}$ separately from the currently growing upper roots, middle roots, and lower roots, the growing upper roots gained N from the older roots, but no net-import of N from the growing upper roots was found in the mature middle and lower roots (Tatsumi and Kono, 1980b). The results indicate that ^15^N taken up by the middle and lower roots first accumulated in the shoots and re-translocated to the growing roots. Transport of the shoot N, which had been labeled with ^15^N, to the roots (proteins) was also observed in the soybean plants at their vegetative growth stage (Yoneyama and Ishizuka, 1982).

### Distribution of N and ^15^N in the Whole Plant

A terminal mature leaf blade of the primary stem was fed at the early booting with ^15^N-labeled NO_2_ (which can be assimilated to AAs in leaf cells) for 2 h and ^15^N partitioning was followed in the whole plant over 8 days. ^15^N was actively transported to the growing panicles, tillers, and middle and upper roots during the first day and gradually increased during the subsequent days, whereas the ^15^N partition to the other mature leaves was not significant (Okano et al., 1983).

**FIGURE 5:**
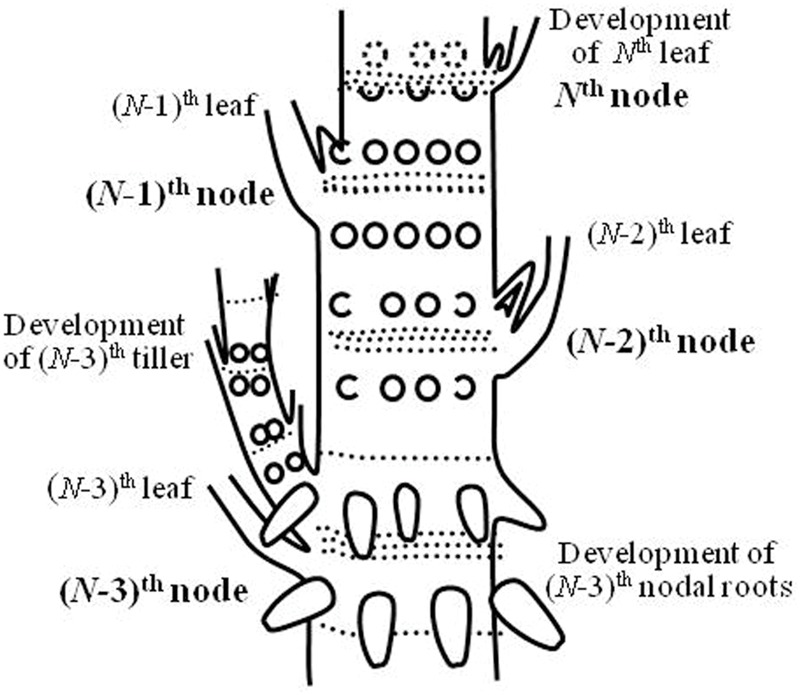
**Synchronized growth of leaves, tillers, and roots.** When the *N*th leaf blade develops from the *N*th node, the tiller and roots actively develop from the (*N*-3)th node. This image was drawn from the Japanese edition of Hoshikawa (1981).

As stated above, a substantial part (∼50%) of N in the differentiating and growing organs (new leaves, tillers, roots, and differentiating panicles) may be supplied by remobilization of the reserved N during the vegetative growth and early reproductive development (i.e., at spikelet primordia differentiation). The meristems in the growing organs may receive AAs via phloem after remobilization of the reserves accumulated in mature leaves, stems, and roots as proteins, nucleic acids, free AAs, etc., with the remainder being supplemented via xylem-to-phloem transfer of the xylem-ascending AAs produced from the current root uptake of ammonium and nitrate. It is interesting to note that when the *N*th leaf blade is growing, new roots and a tiller grow from the (*N-*3)th node (**Figure 5**), and such synchronized growth of the leaves, tillers, (Katayama, 1951; Jaffuel and Dauzat, 2005) and roots is probably replenished by the N reserves of the (*N-*1)th, (*N-*2)th, and (*N-*3)th leaves, and additional re-mobilized N comes from matured leaves and roots located in nodes lower than (*N-*4)th.

Demands for new growth constitute sink activity, and such demands must be transferred successively. For example, systematic differentiation of new leaves one-by-one at the meristems must be supported by the stable supply of the required AAs (Yoneyama, 1978a) to synthesize nucleic acids and proteins and also by the supply of carbohydrates to produce energy (ATP) and reductants (NADH, NADPH). To satisfy the progressive demand, the internal resources in mature organs should be ready for transport as low-molecular-mass compounds through the phloem after remobilization of the high-molecular-mass N compounds and carbohydrates in mature organs.

The major N-containing low-molecular-mass compounds transported from the reserves via phloem are AAs re-mobilized from mature leaves and to a lesser extent from stems and roots. In leaves, they are produced by nitrate reduction, ammonia assimilation and protein turnover (synthesis and degradation). The re-mobilized N is derived mainly from rapidly degraded proteins in the cytosol of the cells through highly regulated intracellular systems including the proteasome system (Yanagawa et al., 1999; Nelson et al., 2014) and supplemented partly from leaf organelle-localized protein pools such as Rubisco in the chloroplasts through autophagy pathway (Nelson et al., 2014; Wada et al., 2015), as well as from leaf structural and membrane proteins and stem storage proteins. The contribution of AAs derived from storage proteins, which constitute a synthesis and degradation system, was demonstrated during the new leaf growth in perennial ryegrass (*Lolium perenne*) (Lehmeier et al., 2013).

**FIGURE 6:**
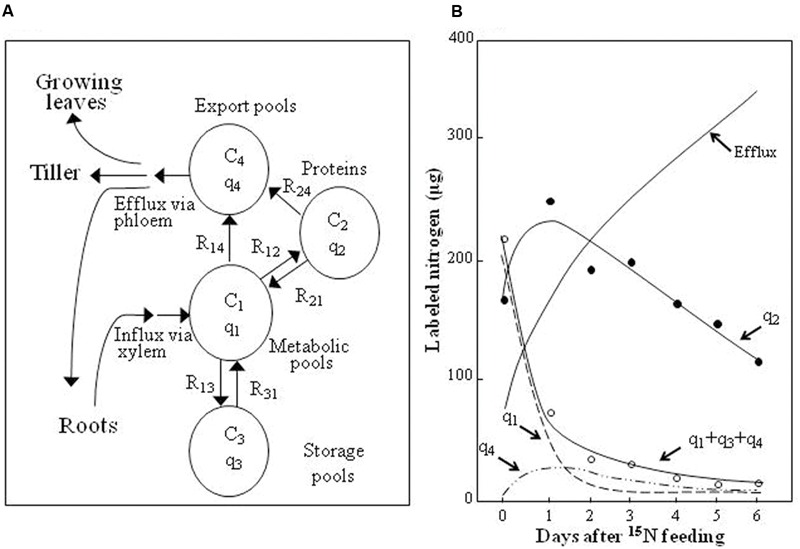
**(A)** Four-compartment model including four different types of N pools (C), quantities of labeled N (q) and transfer rates (R) between compartments in mature rice leaves. **(B)** Simulated partitions of labeled N in different pools in the middle leaves of rice. Adapted from Yoneyama and Takeba (1984).

The fluxes of N through four compartments in the mature rice leaf are shown in **Figure 6A**. AAs and nitrate enter the metabolic pools (Compartment 1) via xylem to synthesize proteins (Compartment 2), although some N in the metabolic pools may be transferred to the temporary storage pools (Compartment 3) or directly to the export pools (Compartment 4). The N exported may reach the growing leaves, tillers and roots. This four-compartment model was applied to simulate the fluxes of pulsed ^15^N in mature rice leaves (**Figure 6B**, Yoneyama and Takeba, 1984). The results of that simulation indicated that ^15^N was distributed in four compartments with appropriate N transfer rates (*R* values). Regarding the eﬄux of N from the mature leaves, three important processes were found: (1) the eﬄux of N has two origins, one through the protein compartment (*R*_24_) and the other directly from the metabolic pools (*R*_14_) with the amount of *R*_14_ being 30% of *R*_24_; (2) proteins, including soluble Rubisco and other proteins and structural and membrane proteins, exhibited turnover. ^15^N in the protein pools showed a continuous decrease with half times (*T*_1/2_) of approximately 4 days in the middle leaves and approximately 6 days in the older lower leaves. The degradation of proteins by proteinases through the proteasome and autophagy pathways produces a large part of the re-mobilized proteinogenic AAs, and (3) the level of reutilization of the re-mobilized AAs for protein synthesis was very small.

The amounts of eﬄux N from four N fractions (free AAs, soluble proteins except Rubisco, Rubisco and structural and membrane proteins) were calculated in **Table 2**. In this calculation, the relative N sizes of the four fractions at the maximum N accumulation (Mae et al., 1983) were assumed to decrease linearly to the half values by 28 days, with the respective *T*_1/2_ values estimated for three protein fractions. The *T*_1/2_ values of the soluble proteins except Rubisco fraction were 4 days during 28 days, and those of Rubisco fraction were 4 days for the first 14 days in the just-matured leaf and 8 days for the period between day 15 and 28 in the older leaves. Imai et al. (2005) reported such a slowdown of Rubisco turnover in the 8th leaf at the senescent stage, and suggested that it was probably due to a reduction in N import. The *T*_1/2_ of the structural and membrane protein fraction was assumed to be 28 days, indicating small rates of new synthesis. The amount of N eﬄux was calculated every 7 days for 7, 14 (7 × 2), 21 (7 × 3) and 28 (7 × 4) days. Approximately, every 7 days, a new leaf blade or sheath is developed from or within the previously developed leaf sheath in Japonica-type rice plants (Hoshikawa, 1981). The estimated amounts of N eﬄux through the whole leaf and the protein pool, which may release proteinogenic AAs, during the first 7 days (76.2 and 58.6 units, respectively) were larger than the estimated amounts of net decrease of leaf N (100/4/2 or 12.5 units) and protein pool N (92/4/2 or 11.5 units). By 14 days (equivalent to the whole leaf development), the estimated amounts of N eﬄux through the whole leaf (144 units) and the protein pool (111 units) were larger than those of the initial N of the whole leaf (100 units) and protein pool (92 units). In the older leaves (equivalent to an age of 28 days), a considerable amount of 3N (229 units) had been exported, compared to the net decrease (50 units) in leaf 3N. It is noteworthy that the N eﬄux during the first 14 days, which contained a large amount of proteinogenic AAs (111/144 × 100 or 77%), is translocated via phloem to support the growth of new leaves, tillers, and roots (**Figure 6A**) along with the currently taken up N, which is transported directly via xylem and partly through the xylem-to phloem transfer, as shown in **Figure 2**.

**Table 2:** Temporal changes of N eﬄux from a mature leaf involving protein turnover in leaf N fractions.

		Turnover	
N fraction (source)	N size (unit) at maximum N^a^ 0 day	T_1/2_^b^(days)	Rate^c^(day^-1^)	N eﬄux (unit) for 7 days	N eﬄux (unit) for 14 days	N eﬄux (unit) for 21 days	N eﬄux (unit) for 28 days
Free amino acids	8	(Eﬄux from eﬄux protein) × 0.3		17.6	33.2	43.7	52.9
Soluble proteins except Rubisco	21	4	0.173	23.4	44.6	63.1	79.3
Rubisco	25	4 or 8^d^	0.173 or0.087	27.8	52.2	62.9	72.3
Structural and membrane proteins	46	28	0.025	7.4	13.9	19.6	24.6
Total unit	100			76.2	144	189	229

*The total leaf N was assumed to decrease linearly at the same rates in the four N fractions to half of the maximum after 28 days. ^a^Data from Mae et al. (1983). ^b^Data from Yoneyama and Takeba (1984). ^c^Calculated by division of -ln (0.5) or 0.693 by T_1/2_. ^d^The *T*_1/2_ values of the Rubisco fraction were assumed to be 4 days for the first 14 days and 8 days for the next 14 days (from day 15 and 28).*

Recently it has been demonstrated that the autophagy pathway plays at least a partial role in the degradation of Rubisco in rice leaves during senescence at the vegetative stage (Wada et al., 2015). An autophagy-disrupted rice mutant, *Osatg7-1*, grown in medium with an ample supply of N reduced leaf area and tiller numbers, possibly due to a reduction in the transport of re-mobilized N from Rubisco protein, which could be 37% of total eﬄux at the maximum (**Table 2**). In the second phase of the reproductive stage (**Table 3**), the N reserves in the top four leaves is intensively utilized for grain-filling, and under such circumstances, Rubisco proteins undergo active degradation via autophagy pathway as described in the later section.

**Table 3:** The two phases of the reproductive stage: 32 days before and 50 days after heading at day 0.

Days before and after heading	Events	
	In panicles	In vegetative organs
-32	Differentiation and development of panicle primordia and spikelets in the main stem and tillers.	Development of top four leaves (including flag leaf) by 3–6 days’ intervals and elongation of top four internodes.
(-12)	Flower-organ differentiation.	Small degradation of Rubisco.Accumulation of storage in stems and leaf sheaths.
0	Heading, anthesis and fertilization.	
(+ 4)	Grain filling with proteins and carbohydrates.	Intensive degradation and remobilization of leaf proteins, especially Rubisco and stem storage compounds.
+50	Complete maturing	

## Modeling of the Nitrogen Relation in Two Phases at the Reproductive Stage: Panicle and Spikelet Development and Grain Filling

### N Accumulation during the Panicle Development Is Regulated According to the Type of Vegetative Growth: The First Phase of the Reproductive Stage

The reproductive stage of rice plants consists of two distinct phases (**Table 3**), as proposed by Murayama (1957). The first phase of the reproduction occurs during the 32 days before heading (day 0; emergence of panicles from the flag-leaf sheath): the development of the flag-leaf on the main stem is the 15th, starting by the primary leaf at germination. During the early period (between –32 and –12 days) of the first phase, panicle primordia and spikelets are differentiated and developed in the meristems of the stem top on the necknode, and the top four leaves (including the flag-leaf) and the internodes corresponding to each of the four leaves are successively developed on the mature dwarf stem and leaves (11 leaves from the primary leaf). The panicle primordia and spikelet development and the growth of the top four leaves and internodes are mainly sustained by the N stored in the dwarf stem-attached leaves and supplemented by the newly taken up N from the soil and top-dressed fertilizer. An exogenous supply of N is very important to maintain the number of effective spikelets with a smaller number of degenerated spikelets (Arai and Kono, 1978) since the N requirement for the growth of the top four leaves is very competitive with that of the spikelets. However, an excess application of top-dressing fertilizers (ammonium) causes an increase of the lodging rate, probably due to elongation of the dwarf-stem internode (Takebe et al., 1994).

During the late period (between –12 and 0 days) of the first phase, flowering organs are differentiated and self-fertilization occurs 2 days before heading. During this period, vegetative organs slow growth, whereas the top four leaves are quite active in photosynthesis, and Rubisco proteins are maintained with low degradation rates, and significant amounts of organic N compounds and carbohydrates are stored in stems and leaf sheaths (Yoneyama et al., 1989). These are used subsequently at the rapid events of heading, flowering and pollination, which occur during the 4 days after heading.

Under short-day conditions, a flower-promoting protein, Hd3a (Tamaki et al., 2007), is transported from the leaf blades to the shoot apical meristem during the first phase of the reproductive stage. This phloem signal transport is a key trigger of developmental transfer from the vegetative to reproductive stages.

### N Accumulation in the Grains by Sink Demand: The Second Phase of the Reproductive Stage

The second phase of the reproductive stage (in the last 50 days) is the endosperm development, filling grains with storage proteins and carbohydrates. During grain-filling, 70–90% of grain N is transported from the plant internal reserves in the vegetative organs and the rest (10–30%) is supplemented from the soil and late top-dressed fertilizers (Mae, 2010). Sixty percent of the re-translocated N is derived from the leaf blades of the top four leaves, with two-thirds (31–37%) coming from the degradation of Rubisco (Makino et al., 1984). The remaining 40% of the re-translocated N is from the leaf sheaths, stems, and to a lesser extent from the roots. The autophagic degradation of Rubisco in rice leaves may contribute to an efficient and rapid N remobilization by facilitating protein degradation for N mobilization in senescent leaves. The net rate of Rubisco degradation was faster than that of the remaining soluble proteins (Mae et al., 1983).

It should be noted that the application of ammonia fertilizers at heading increases the contents of storage proteins (glutelin, prolamin) in grains (white rice). The excessive protein contents and an accompanying reduction of carbohydrates (sucrose) lead to a reduction of eating quality (taste) (Takebe et al., 1994, 1996).

When ^15^N-labeled nitrate applied to field-grown corn at post-silking, the applied ^15^N was simultaneously allocated to the stovers and grains. The ^15^N partitioned to the stovers was incorporated into the proteins as a consequence of its turnover, and finally the ^15^N-labeled stover protein was hydrolyzed and its products were transported to the grain (Gallais et al., 2006).

### Molecular Approaches to Increasing Grain Yields

In the scheme shown in **Table 3**, the spikelet numbers are determined by a vegetative growth system in which the growth of new organs is sustained by both of the remobilization from the leaves as well as new root uptake, while the numbers of grains (filled-spikelets) are determined by the grain-filling system accompanying the rapid degradation of Rubisco in the top four leaves.

A quantitative trait locus (QTL) for cytosolic GS1 in rice was revealed to enhance tillering at the vegetative stage and increase the number and weight of panicles (Obara et al., 2004), probably due to enhanced root N assimilation. The cytosolic OsGS1;2 functions in primary ammonia assimilation in the roots: GS1;2-deficient rice lines showed severe N deficiency and decrease of tiller development (Funayama et al., 2013). The importance of leaf-blade-expressed OsGS1;1 was examined in knockout rice plants: both the spikelet numbers and grain filling in the transgenics were reduced (Tabuchi et al., 2005), probably due to the small transport of internal N.

In a transgenic wheat line where leaf GS1 activity was activated, N and dry matter contents in grains as well as in roots were increased, although the grain numbers were not different from those of the non-transgenic control (Habash et al., 2001). In maize, cytosolic GS1;3, localized in leaf mesophyll cells, plays an important role in determining kernel numbers, while GS1;4, localized in bundle sheath cells, is important for increasing kernel size. Over-expression of the GS1;3 in leaves led to a 30% increase in kernel numbers, probably through the efficient transport of AAs via the phloem at kernel filling (Martin et al., 2006).

In *OsNADH-GOGAT1*-knockout mutants, which have shorter roots and less ammonia-assimilation, the number of spikelets, tillers and panicles were all decreased (Tamura et al., 2010), probably due to a shortage of internal AAs. The *OsNADH-GOGAT2* gene is expressed in fully expanded leaf blades and leaf sheaths, and in the *OsNADH-GOGAT2*-knockout mutants, the spikelet (also ripened spikelet) numbers were reduced (Tamura et al., 2011), probably due to the low level of AA phloem transport. Over-expression of the *Japonica*-type Sasanishiki *NADH-GOGAT* gene in the *Indica*-type Karakath line caused an increase of the spikelet weight, but the grain numbers were not changed (Yamaya et al., 2002).

A recent identification in Karakath rice of the *PSTOL1* gene, which is involved in phosphorus (P)-deficiency tolerance in soils, has provided a genetic tool for increasing grain yield, since it can be introduced into P-deficiency-intolerant rice genotypes such as the Japonica type rice (Gamuyao et al., 2012). Indeed, the *PSTOL1*-gene introduction increased the root weight and length and nutrient uptake (P, K, and N). The enhanced uptake of these nutrients due to abundant roots and an efficient nutrient distribution to grains had previously been recorded in high-yielding rice varieties (Murakami and Yoneyama, 1988; Yoneyama et al., 1989).

## Conclusion

Rice plants transport specific AAs, derived from internal remobilization and new uptake and assimilation, to the shoot and root meristems to produce new organs and tissues for vegetative growth, and such N is also imported to carry out anthesis and produce embryos in the early reproductive stage. Rice plants produce grain endosperms, whose storage compounds such as proteins and starch are for the next generation, largely by collecting internally available resources from the top four leaves and the stems through protein remobilization. Thus, the efficient formation of “350 grains” may be predominantly sustained by the formation of reserves during 100 days in different leaves, tillers and roots through unique growth and N-allocation systems, and finally through a 50-day remobilization period when the AAs in such reserves are re-allocated. This review provides the first dynamic description of these processes in rice.

## Author Contributions

TY wrote the first draft of the manuscript and organized the tables and figures. FT, JT, and TM reviewed the manuscript and added information.

## Conflict of Interest Statement

The authors declare that the research was conducted in the absence of any commercial or financial relationships that could be construed as a potential conflict of interest.

The reviewer SK declared a shared affiliation, though no other collaboration, with one of the authors TM to the handling Editor, who ensured that the process nevertheless met the standards of a fair and objective review.

## References

[B1] Arai K. Kono Y. (1978). Development of the rice panicle. I. Characteristics of the growth of spikelets at different positions on panicle. *Jpn. J. Crop Sci.* 47 699–706. 10.1626/jcs.47.699

[B2] Britto D. T. Kronzucker H. J. (2004). Bioengineering nitrogen acquisition in rice: can novel initiatives in rice genomics and physiology contribute to global food security? *Bioessays* 26 683–692. 10.1002/bies.20040 15170866

[B3] Fukumorita T. Chino M. (1982). Sugar, amino acid and inorganic contents in rice phloem sap. *Plant Cell Physiol.* 23 273–283.

[B4] Funayama K. Kojima S. Tabuchi-Kobayashi M. Sawa Y. Nakayama Y. Hayakawa T. (2013). Cytosolic glutamine synthetase 1;2 is responsible for the primary assimilation of ammonium in rice roots. *Plant Cell Physiol.* 54 934–943. 10.1093/pcp/pct046 23509111

[B5] Gallais A. Coque M. Quilléré I. Prioul J.-L. Hirel B. (2006). Modelling postsilking nitrogen fluxes in maize (*Zea mays*) using 15N-labelling field experiments. *New Phytol.* 172 696–707. 10.1111/j.1469-8137.2006.01890.x 17096795

[B6] Gamuyao R. Chin J. H. Pariasca-Tanaka J. Pesaresi P. Catausan S. Dalid C. (2012). The protein kinase Pstoll from traditional rice confers tolerance of phosphorus deficiency. *Nature* 488 535–539. 10.1038/nature11346 22914168

[B7] Habash D. Z. Massiah A. J. Rong H. L. Wallsgrove R. M. Leigh R. A. (2001). The role of cytosolic glutamine synthetase in wheat. *Ann. Appl. Biol.* 138 83–89. 10.1111/j.1744-7348.2001.tb00087.x

[B8] Hasegawa H. (1996). Selection for mutants with low nitrate uptake ability in rice (*Oryza sativa*). *Physiol. Plant.* 96 199–204. 10.1034/j.1399-3054.1996.960205.x

[B9] Hayakawa K. Yamaya T. Mae T. Ojima K. (1993). Changes in the content of two glutamate synthase proteins in spikelets of rice (*Oryza sativa*) plants during ripening. *Plant Physiol.* 101 1257–1262. 12231780 10.1104/pp.101.4.1257 PMC160647

[B10] Hayakawa T. Hopkins L. Peat L. J. Yamaya T. Tobin A. K. (1999). Quantitative intercellular localization of NADH-dependent glutamate synthase protein in different types of root cells in rice plants. *Plant Physiol.* 119 409–416. 10.1104/pp.119.2.409 9952435 PMC32116

[B11] Hayashi H. Chino M. (1985). Nitrate and other anions in the rice phloem sap. *Plant Cell Physiol.* 26 325–330. 10.1093/jxb/eru425 9787465

[B12] Hayashi H. Chino M. (1990). Chemical composition of phloem sap from the uppermost internode of the rice plant. *Plant Cell Physiol.* 31 247–251.

[B13] Hirel B. Gadal P. (1980). Glutamine synthetase in rice. A comparative study of the enzymes from roots and leaves. *Plant Physiol.* 66 619–623. 10.1104/pp.66.4.619 16661490 PMC440691

[B14] Hoshikawa K. (1981). “Morphology and development of the rice plant,” in *Theory of Rice Cultivation and Fundamental Physiology, Rice* Vol. 1 (Tokyo: Nousan-Gyoson-Bunka Association) 419–431.

[B15] Hsu P.-K. Tsay Y.-F. (2013). Two phloem nitrate transporters, NRT1.11 and NRT1.12, are important for redistributing xylem-bone nitrate to enhance plant growth. *Plant Physiol.* 163 844–856. 10.1104/pp.113.226563 24006285 PMC3793062

[B16] Imai K. Suzuki Y. Makino A. Mae T. (2005). Effects of nitrogen nutrition on the relationships between the levels of rbcS and rbcL mRNAs and the amount of ribulose 1⋅5-bisphosphate carboxylase/oxygenase synthesized in the eighth leaves of rice from emergence through senescence. *Plant Cell Environ.* 28 1589–1600. 10.1111/j.1365-3040.2005.01438.x

[B17] Jaffuel S. Dauzat J. (2005). Synchronism of leaf and tiller emergency relative to position and to main stem development stage in a rice cultivar. *Ann. Bot.* 95 401–412. 10.1093/aob/mci043 15601682 PMC4246790

[B18] Kamachi K. Yamaya T. Hayakawa T. Mae T. Ojima K. (1992). Vascular bundle-specific localization of cytosolic glutamine synthetase in rice leaves. *Plant Physiol.* 99 1481–1486. 10.1104/pp.99.4.1481 16669062 PMC1080651

[B19] Kamada-Nobusada T. Makita N. Kojima M. Sakakibara H. (2013). Nitrogen-dependent regulation of de novo cytokinin biosynthesis in rice: the role of glutamine metabolism as an additional signal. *Plant Cell Physiol.* 54 1881–1893. 10.1093/pcp/pct127 24058148 PMC3814184

[B20] Katayama T. (1951). *Studies on Tillering of Rice, Wheat and Barley.* Tokyo: Yokendo.

[B21] Kawachi T. Sueyoshi K. Nakajima A. Yamagata H. Sugimoto T. Oji Y. (2002). Expression of asparagine synthetase in rice (*Oryza sativa*) roots in response to nitrogen. *Physiol. Plant.* 114 41–46. 10.1034/j.1399-3054.2002.1140107.x 11982933

[B22] Kawahara H. Chonan N. Matsuda T. (1974). Studies on morphogenesis in rice plants. 7. The morphology of vascular bundles in the vegetative nodes of the culm. *Jpn. J. Crop Sci.* 43 389–401. 10.1626/jcs.43.389

[B23] Kiyomia S. Nakanishi H. Uchida H. Tsuji A. Nishiyama S. Futatsubashi M. (2001). Real time visualization of 13N-translocation in rice under different environmental conditions using positron emitting trace imaging system. *Plant Physiol.* 125 1743–1753. 10.1104/pp.125.4.1743 11299355 PMC88831

[B24] Lawrence J. M. Grant D. R. (1964). Incorporation of lysine-14C into the developing grain of wheat. *Arch. Biochem. Biophys.* 104 73–78. 10.1016/S0003-9861(64)80036-9

[B25] Lehmeier C. A. Wild M. Schnyder H. (2013). Nitrogen stress affects the turnover and size of nitrogen pools supplying leaf growth in a grass. *Plant Physiol.* 162 2095–2105. 10.1104/pp.113.219311 23757403 PMC3729785

[B26] Lin C.-M. Koh S. Stacey G. Yu S.-M. Lin T.-Y. Tsay Y.-F. (2000). Cloning and functional characterization of a constitutively expressed nitrate transporter gene, OsNRT1, from rice. *Plant Physiol.* 122 379–388. 10.1104/pp.122.2.379 10677431 PMC58875

[B27] Mae T. (2010). “Nitrogen utilization, growth and yield in rice plants,” in *Nitrogen Assimilation in Plants* eds Ohyama T. Sueyoshi K. (Kerala: Research Signpost) 243–253.

[B28] Mae T. (2011). Nitrogen acquisition and its relation to growth and yield in recent high-yielding cultivars of rice (*Oryza sativa* L.) in Japan. *Soil Sci. Plant Nutr.* 57 625–635. 10.1080/00380768.2011.602626

[B29] Mae T. Makino A. Ohira K. (1983). Changes in the amounts of ribulose bisphosphate carboxylase synthesized and degraded during the life span of rice leaf (*Oryza sativa* L.). *Plant Cell Physiol.* 24 1079–1086.

[B30] Mae T. Ohira K. (1981). The remobilization of nitrogen related to leaf growth and senescence in rice plants (*Oryza sativa* L.). *Plant Cell Physiol.* 22 1067–1074.

[B31] Makino A. Mae T. Ohira K. (1984). Relation between nitrogen and ribulose-1,5-bisphosphate carboxylase in rice leaves from emergence through senescence. *Plant Cell Physiol.* 25 429–437.

[B32] Martin A. Lee J. Kichey T. Gerentes D. Zivy M. Tatout C. (2006). Two cytosolic glutamine synthetase isoforms of maize are specifically involved in the control of grain production. *Plant Cell* 18 3252–3274. 10.1105/tpc.106.042689 17138698 PMC1693956

[B33] Mimoto H. Hattori M. Chujo H. (1990). Translocation of nitrogen absorbed by the roots of specific tiller in rice plant. *Jpn. J. Crop Sci.* 59 369–376. 10.1626/jcs.59.369

[B34] Murakami T. Yoneyama T. (1988). Comparison of root length of two rice (*Oryza sativa* L.) varieties by using an image analyzer. *Plant Soil* 105 287–289. 10.1007/BF02376794

[B35] Murayama N. (1957). Studies on nitrogen metabolism of the rice plant in relationship to its growth. *Soil Plant Food* 2 134–141. 10.1080/00380768.1956.10431873

[B36] Nelson C. J. Li L. Millar A. H. (2014). Quantitative analysis of protein turnover in plants. *Proteomics* 14 579–592. 10.1002/pmic.201300240 24323582

[B37] Noctor G. Novitskaya L. Lea P. J. Foyer C. H. (2002). Co-ordination of leaf minor amino acid contents in crop species: significance and interpretation. *J. Exp. Bot.* 53 939–945. 10.1093/jexbot/53.370.939 11912236

[B38] Obara M. Sato T. Sasaki S. Kashiba K. Nagano A. Nakamura I. (2004). Identification and characterization of a QTL on chromosome 2 for cytosolic glutamine synthetase content and panicle number in rice. *Theor. Appl. Genet.* 110 1–11. 10.1007/s00122-004-1828-0 15549232

[B39] Ohashi M. Ishiyama K. Kojima S. Konishi N. Nakano K. Kanno K. (2015). Asparagine synthetase1, but not asparagine synthetase2, is responsible for the biosynthesis of asparagine following the supply of ammonium to rice roots. *Plant Cell Physiol.* 56 769–778. 10.1093/pcp/pcv005 25634963

[B40] Okano K. Tatsumi J. Yoneyama T. Kono Y. Totsuka T. (1983). Investigation on the carbon and nitrogen transfer from a terminal leaf to the root system of rice plant by a double tracer method with 13C and 15N. *Jpn. J. Crop Sci.* 52 331–341. 10.1626/jcs.52.331

[B41] Peterson D. M. Housley T. L. Schrader L. E. (1977). Long distance translocation of sucrose, serine, leucine, lysine, and CO2 assimilates. II. Oats. *Plant Physiol.* 59 221–224. 10.1104/pp.59.2.221 16659821 PMC542369

[B42] Pratelli R. Pilot G. (2014). Regulation of amino acid metabolic enzymes and transporters in plants. *J. Exp. Bot.* 65 5535–5556. 10.1093/jxb/eru320 25114014

[B43] Shewry P. R. Halford N. G. (2002). Cereal seed storage proteins: structures, properties and role in grain utilization. *J. Exp. Bot.* 53 947–958. 10.1093/jexbot/53.370.947 11912237

[B44] Sonoda Y. Ikeda A. Saiki S. von Wirén N. Yamaya T. Yamaguchi J. (2003). Distinct expression and function of three ammonium transporter genes (OsAMT1;1–1;3) in rice. *Plant Cell Physiol.* 44 726–734. 10.1093/pcp/pcg083 12881500

[B45] Suzuki A. Vidal J. Gadal P. (1982). Glutamate synthase isoforms in rice. Immunological studies of enzymes in green leaf, etiolated leaf, and root tissues. *Plant Physiol.* 70 827–832. 10.1104/pp.70.3.827 16662583 PMC1065778

[B46] Tabuchi M. Sugiyama K. Ishiyama K. Inoue E. Sato T. Takahashi H. (2005). Severe reduction in growth rate and grain filling of rice mutants lacking OsGS1;1, a cytosolic glutamine synthetase1;1. *Plant J.* 42 641–651. 10.1111/j.1365-313X.2005.02406.x 15918879

[B47] Takebe M. Miyata K. Kanamura N. Yoneyama T. (1994). Changes in contents of sugars and amino acids in brown rice during grain ripening and influence of nitrogen treatments. *Jpn. J. Soil Sci. Plant Nutr.* 65 503–513.

[B48] Takebe M. Oikawa T. Matsuno K. Shimizu E. Yoneyama T. (1996). Influence of nitrogen application on the contents of glutelin and prolamin of polished rice grains (*Oryza sativa* L.). *Jpn. J. Soil Sci. Plant Nutr.* 67 139–146.

[B49] Tamaki S. Matsuo S. Wong H. L. Yokoi S. Shimamoto K. (2007). Hd3a protein is a mobile flowering signal in rice. *Science* 316 1033–1036. 10.1126/science.1141753 17446351

[B50] Tamura W. Hidaka Y. Tabuchi M. Kojima S. Hayakawa T. Sato T. (2010). Reverse genetics approach to characterize a function of NADH-glutamate synthase1 in rice plants. *Amino Acids* 39 1003–1012. 10.1007/s00726-010-0531-5 20213442

[B51] Tamura W. Kojima S. Toyokawa A. Watanabe H. Tabuchi-Kobayashi M. Hayakawa T. (2011). Disruption of a novel NADH-glutamate synthase2 gene caused marked reduction in spikelet number of rice. *Front. Plant Sci.* 2:57 10.3389/fpls.2011.00057 PMC3355815 22645542

[B52] Tanaka A. Garcia C. V. (1965). Studies of the relationship between tillering and nitrogen uptake of the rice plant. *Soil Sci. Plant Nutr.* 11 31–37. 10.1080/00380768.1965.10431146

[B53] Tanaka T. Suzui N. Hayashi H. Yamaya T. Yoneyama T. (2009). Cytosolic glutamine synthetase is present in the phloem sap of rice (*Oryza sativa* L.). *Soil Sci. Plant Nutr.* 55 102–106. 10.1111/j.1747-0765.2008.00327.x

[B54] Tatsumi J. Kono Y. (1980a). Root growth of rice plants in relation to nitrogen supply from shoot. *Jpn. J. Crop Sci.* 49 112–119. 10.1104/pp.112.204461

[B55] Tatsumi J. Kono Y. (1980b). Nitrogen uptake and transport by the intact root system of rice plants: comparison of the activity in roots from different nodes. *Jpn. J. Crop Sci.* 49 349–358. 10.1626/jcs.49.349

[B56] Tatsumi J. Kono Y. (1981). Translocation of foliar-applied nitrogen to rice roots. *Jpn. J. Crop Sci.* 50 302–310. 10.1626/jcs.50.302

[B57] Taylor M. R. Reinders A. Ward J. M. (2015). Transport function of rice amino acid permeases (AAPs). *Plant Cell Physiol.* 56 1355–1363. 10.1093/pcp/pcv053 25907566

[B58] Tegeder M. Rentsch D. (2010). Uptake and partitioning of amino acids and peptides. *Mol. Plant* 3 997–1011. 10.1093/mp/ssq047 21081651

[B59] Tegeder M. Ward J. M. (2012). Molecular evolution of plant AAP and LHT amino acid transporters. *Front. Plant Sci.* 3:21 10.3389/fpls.2012.00021 PMC3355764 22645574

[B60] van Bel A. J. E. (1984). Quantification of the xylem-to-phloem transfer of amino acids by use of inulin [14C]carboxylic acid as xylem transport marker. *Plant Sci. Lett.* 35 81–85. 10.1016/0304-4211(84)90162-7

[B61] Wada S. Hayashida Y. Izumi M. Kurusu T. Hanamata S. Kanno K. (2015). Autophagy supports biomass production and nitrogen use efficiency at the vegetative stage in rice. *Plant Physiol.* 168 60–73. 10.1104/pp.15.00242 25786829 PMC4424030

[B62] Yamaya T. Hayakawa T. Tanasawa K. Kamachi K. Mae T. Ojima K. (1992). Tissue distribution of glutamate synthase and glutamine synthetase in rice leaves. Occurrence of NADH-dependent glutamate synthase protein and activity in the unexpanded, nongreen leaf blades. *Plant Physiol.* 100 1427–1432. 10.1104/pp.100.3.1427 16653141 PMC1075802

[B63] Yamaya T. Obara M. Nakajima H. Sasaki S. Hayakawa T. Sato T. (2002). Genetic manipulation and quantitative-trait loci mapping for nitrogen recycling in rice. *J. Exp. Bot.* 53 917–925. 10.1093/jexbot/53.370.917 11912234

[B64] Yamaya T. Tanno H. Hirose N. Watanabe S. Hayakawa T. (1995). A supply of nitrogen causes increase in the level of NADH-dependent glutamate synthase protein and in the activity of the enzyme in roots of rice seedlings. *Plant Cell Physiol.* 36 1197–1204.

[B65] Yanagawa Y. Ohhashi A. Murakami Y. Saeki Y. Yokosawa H. Tanaka K. (1999). Purification and characterization of the 26S proteasome from culture rice (*Oryza sativa*) cells. *Plant Sci.* 149 33–41. 10.1016/S0168-9452(99)00140-5

[B66] Yoneyama T. (1977). Nitrogen nutrition and growth of the rice plant. I. Nitrogen circulation and protein turnover in rice seedlings. *Soil Sci. Plant Nutr.* 23 237–245. 10.1080/00380768.1977.10433041

[B67] Yoneyama T. (1978a). Nitrogen nutrition and growth of the rice plant. III. Origin of amino-acid nitrogen in the developing leaf. *Soil Sci. Plant Nutr.* 24 199–205. 10.1080/00380768.1978.10433096

[B68] Yoneyama T. (1978b). Utilization of seed and medium nitrogen in young plant seedlings. *Soil Sci. Plant Nutr.* 24 289–293. 10.1080/00380768.1978.10433104

[B69] Yoneyama T. (1983). Distribution of nitrogen absorbed during different times of growth in the plant parts of wheat and contribution to the grain amino acids. *Soil Sci. Plant Nutr.* 29 193–207. 10.1080/00380768.1983.10432420

[B70] Yoneyama T. (1986). Absorption and assimilation of nitrogen by rice plants. A review on 15N study in Japan. *JARQ* 20 121–126.

[B71] Yoneyama T. Fukuda M. Kouchi H. (1989). Partitioning of carbon, nitrogen, phosphorus, potassium, calcium, and magnesium in a semidwarf high-yielding rice variety: comparison with a conventional japonica variety. *Soil Sci. Plant Nutr.* 35 43–54. 10.1080/00380768.1989.10434735

[B72] Yoneyama T. Ishizuka J. (1982). 15N study on the partitioning of the nitrogen taken by soybeans from atmospheric dinitrogen, medium nitrate and ammonium. *Soil Sci. Plant Nutr.* 28 451–461. 10.1080/00380768.1982.10432385

[B73] Yoneyama T. Ito O. Engelaar W. M. H. G. (2003). Uptake, metabolism and distribution of nitrogen in crop plants traced by enriched and natural 15N: progress over the last 30 years. *Phytochem. Rev.* 2 121–132. 10.1023/B:PHYT.0000004198.95836.ad

[B74] Yoneyama T. Kumazawa K. (1972). Differences in the distribution patterns of 15NO3-N and 15NH4-N in rice seedlings. *Jpn. J. Soil Sci. Plant Nutr.* 43 329–332.

[B75] Yoneyama T. Kumazawa K. (1974). A kinetic study of the assimilation of 15N-labelled ammonium in rice seedling roots. *Plant Cell Physiol.* 15 655–661.

[B76] Yoneyama T. Kumazawa K. (1975). A kinetic study of the assimilation of 15N-labelled nitrate in rice seedlings. *Plant Cell Physiol.* 16 21–26.

[B77] Yoneyama T. Sano C. (1978). Nitrogen nutrition and growth of the rice plant. II. Considerations concerning the dynamics of nitrogen in rice seedlings. *Soil Sci. Plant Nutr.* 24 191–198. 10.1080/00380768.1978.10433095

[B78] Yoneyama T. Takeba G. (1984). Compartment analysis of nitrogen flows through mature leaves. *Plant Cell Physiol.* 25 39–48.

[B79] Zee S.-Y. (1972). Transfer cells and vascular tissue distribution in the vegetative nodes of rice. *Aust. J. Bot.* 20 41–48. 10.1071/BT9720041

